# Using a Single VCSEL Source Employing OFDM Downstream Signal and Remodulated OOK Upstream Signal for Bi-directional Visible Light Communications

**DOI:** 10.1038/s41598-017-15856-x

**Published:** 2017-11-20

**Authors:** Chien-Hung Yeh, Liang-Yu Wei, Chi-Wai Chow

**Affiliations:** 1 Department of Photonics, Feng Chia University, Taichung, 40724 Taiwan; 2 Department of Photonics and Institute of Electro-Optical Engineering, National Chiao Tung University, Hsinchu, 30010 Taiwan; 3 Department of Electronic Engineering, The Chinese University of Hong Kong, Hong Kong, China

## Abstract

In this work, we propose and demonstrate for the first time up to our knowledge, using a 682 nm visible vertical-cavity surface-emitting laser (VCSEL) applied in a bi-directional wavelength remodulated VLC system with a free space transmission distance of 3 m. To achieve a high VLC downstream traffic, spectral efficient orthogonal-frequency-division-multiplexing quadrature-amplitude-modulation (OFDM-QAM) with bit and power loading algorithms are applied on the VCSEL in the central office (CO). The OFDM downstream wavelength is remodulated by an acousto-optic modulator (AOM) with OOK modulation to produce the upstream traffic in the client side. Hence, only a single VCSEL laser is needed for the proposed bi-directional VLC system, achieving 10.6 Gbit/s OFDM downstream and 2 Mbit/s remodulated OOK upstream simultaneously. For the proposed system, as a single laser source with wavelength remodulation is used, the laser wavelength and temperature managements at the client side are not needed; and the whole system could be cost effective and energy efficient.

## Introduction

Recently, the bandwidth demands of broadband wireless services are increasing exponentially. However, the available radio frequency (RF) bands are limited and it is unlikely that significant new spectrum can be made available for the wireless communications in the near future^1,2^. One promising option is to utilize the free and unlicensed infrared (IR) and visible light spectra leading to optical wireless communication (OWC). As IR is invisible to human beings, the transmitted power used in IR communication should be limited to prevent damages to human eyes. Hence, IR wireless communication is mainly used in very low data rate wireless communication due to the very limited signal-to-noise (SNR) of the transmitted signal. OWC using the visible light spectrum, known as the visible light communication (VLC) has received considerable attenuations recently due to high transmission data rate^3–7^, high secure and directional transmissions^8–10^. VLC can also deliver other unique wireless applications, such as utilizing in underwater^11,12^, high precision indoor-positioning and navigation^13,14^, and in electromagnetic interference (EMI) prohibited areas, such as in aircrafts and hospital operation rooms. VLC is also regarded as a promising solution for the next generation 5 G mobile communications^15^.

White light-emitting diode (LED) based VLC would restrict the modulation bandwidth to a few MHz due to the long relaxation time of phosphor layer^16,17^. Hence, using high spectral-efficiency modulation schemes^18,19^, multi-input-multi-output (MIMO) technique^20,21^, special LED devices^22^ and R/G/B LED^23^ could enhance significantly the VLC data rate to a few Gbit/s. In order to achieve a higher VLC traffic data rate, the visible LD could be utilized to provide high modulation speed and high electrical-to-optical (EO) conversion efficiency. In recent years, many studies of LD-based VLC have been demonstrated. In 2008, Hanson *et al*. used a 532 nm LD to reach 1 Gbit/s underwater VLC through 2 m transmission length^24^. In 2013, Watson *et al*. demonstrated a 2.5 Gbit/s on-off-keying (OOK) VLC scheme by employing a GaN blue LD in a free space transmission^25^. In 2016, Yeh *et al*. first proposed using 682 nm vertical-cavity surface-emitting laser (VCSEL) to achieve 0.52 to11.86 Gbit/s power-sharing VLC transmission by employing orthogonal-frequency-division-multiplexing quadrature-amplitude-modulation (OFDM-QAM) with bit-power-loading^26^. A 8.148 Gbit/s bi-directional VLC using VCSEL was also reported^27^. Besides, Wu *et al*. also investigated tricolor R/G/B LD for white-lighting VLC with > 8 Gbit/s traffic rate in 2017^28^. However, these reported LD-based VLC systems only demonstrated a single directional transmission without the mentioning of how to provide upstream traffic in a bi-directional transmission. A fiber-based optical wireless communication using downstream on-off-keying (OOK) signal generated by a distributed feedback laser diode (DFB-LD) and Mach-Zehnder modulator (MZM) in the central office and upstream remodulated OOK signal generated by semiconductor optical amplifiers (SOAs) in the mobile device was demonstrated^29^; however, this scheme is operated in the 1.5 µm infra-red (IR) wavelength region; and external modulation is used at the central office. Free-space optical data transmissions using multiple quantum well modulating retro-reflector demonstrated by Naval Research Laboratory (NRL)^30,31^ can produce modulation rates of Mbit/s. However, the remodulation schemes discussed^29–31^ are operated in the IR region. In the future optical wireless network, the bandwidth demand of each user will beyond Gbit/s. However, the LED-based VLC system cannot provide the high enough data rate for end-user^32^; hence laser-based VLC system can suit the purpose. It can provide high data rate and long distance transmission. One application of the laser-based VLC system is the ground-to-train data transmission providing a high speed traffic with transmission distance of 200 m^33^.

In this work, we propose and demonstrate using a 682 nm visible VCSEL laser with 1 GHz modulation bandwidth applied in a bi-directional wavelength remodulated VLC system with a free space transmission distance of 3 m. To achieve a high VLC downstream traffic, spectral efficient OFDM-QAM with bit and power loading algorithms are applied on the VCSEL via a bias-tee (BT) for direct modulation in the central office (CO). In the proposed bi-directional VLC system, the OFDM downstream wavelength can also be remodulated by employing an acousto-optic modulator (AOM) with OOK modulation to produce the upstream traffic in the client side. Hence, only a single VCSEL laser is needed for the proposed bi-directional VLC system, achieving 10.6 Gbit/s OFDM downstream and 2 Mbit/s remodulated OOK upstream simultaneously. As a single laser source with wavelength remodulation is used, the laser wavelength and temperature managements at the client side are not needed; and the whole system could be cost effective and energy efficient.

## Results

### OFDM Downstream Traffic

As mentioned before, in the proposed bi-directional VLC system, the downstream traffic is based on OFDM-QAM modulation, and the upstream traffic is based on a wavelength remodulated OOK modulation. For the downstream VLC signal, the OFDM-QAM modulation with bit-loading and power-loading algorisms are used to enhance the bandwidth efficiency for VLC transmission. Here, the 682 nm visible VCSEL laser is operated at 3.5 mA. In this measurement, the electrical OFDM signal is generated by using home-made MATLAB® program. The generation of OFDM modulation contains the serial-to-parallel (S/P), QAM symbol mapping, parallel-to-serial (P/S), inverse fast Fourier transform (IFFT), cyclic prefix (CP) insertion, and digital-to-analog (DA) conversion. The DA conversion is implemented by an arbitrary waveform generator (AWG, Tektronix® AWG7122) with 6 GSample/s sampling rate. The fast-Fourier transform (FFT) size, CP length and OFDM subcarrier number are 256, 3.03% and 80 respectively.

According to the available modulation bandwidth of the VCSEL laser, 80 OFDM subcarriers are applied to occupy within the frequency of 1.895 GHz. Due to the 1 GHz available modulation bandwidth of the VCSEL, the electrical OFDM signal can dynamically adjust and allocate the signal bandwidth according to the channel response. After a free-space transmission of 3 m, the VLC downstream signal can be received and converted to electrical signal using a 1.25 GHz PIN-based photodiode (PD) in the client side. Then, the OFDM signal is connected to real-time oscilloscope (RTO, Tektronix® CSA 7404) with the sampling rate and analog-to-digital (A/D) conversion resolution of 10 GSample/s and 8 bits respectively.

**Figure 1:**
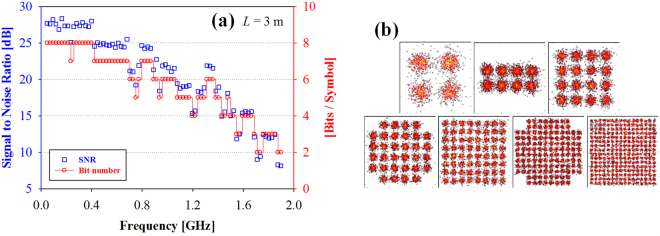
Observed SNR and bit number of each OFDM subcarrier within the modulation frequency of 1.898 GHz in a free space transmission length of 3 m. (**b**) Measured corresponding constellation diagrams of OFDM modulation with bit-power-loading algorithm.

Figure 1(a) shows measured SNR of each OFDM subcarrier within the modulation frequency of 1.898 GHz in a free space transmission length of 3 m. The channel spacing of each OFDM symbol is ~0.024 GHz. As shown in Fig. 1(a), the retrieved SNR decreases at the higher frequency parts. It is due to the power fading and bandwidth limitation of the PIN PD. To achieve the SNR of >16 dB, the corresponding frequency must be within 1.172 GHz. Besides, the entire measured SNRs are between 8.2 and 28.3 dB. To enlarge and optimize the spectral-efficiency of OFDM modulation, the bit and power loading algorithms are designed to use in OFDM channel. Then, the QAM-order can be adapted properly based on the measured SNR of each OFDM subcarrier. Hence, Fig. 1(a) also displays the corresponding number of bit per OFDM symbol in the frequency range of 0.047 to 1.898 GHz after 3 m transmission length. In the measurement, the 4-QAM to 256-QAM OFDM channels can be utilized in the proposed VCSEL VLC system. As seen in Fig. 1(a), the available bit per symbol are from 2−8 bit/sec/Hz. Moreover, Fig. 1(b) shows the corresponding constellation diagrams of OFDM modulation with bit-power-loading algorithm using the 4-, 8-, 16-, 32-, 64-, 128- and 256-QAM formats, respectively, in a free space transmission length of 3 m, respectively. These measured constellations are clear and condensed.

**Figure 2:**
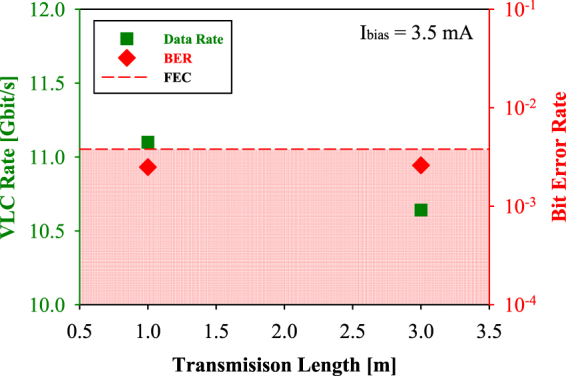
Related VLC data rates and BERs in the free space transmission lengths of 1 and 3 m, respectively.

According to measured SNR of each OFDM subcarrier, the corresponding bit error rate (BER) can be calculated from the error vector magnitude (EVM). Hence, Fig. 2 shows the VLC data rates and BERs in the free space transmission lengths of 1 and 3 m, respectively. The achieved VLC downstream data rates can reach 11.1 and 10.6 Gbit/s, respectively, in the transmission lengths of 1 and 3 m. Moreover, the corresponding BERs are 2.5 × 10^−3^ and 2.6 × 10^−3^ when the transmission lengths are 1 and 3 m, respectively. The whole measured BERs are below the forward error correction (FEC) threshold (BER = 3.8 × 10^−3^). In the measurement, to achieve > 10 Gbit/s downstream data rate in the proposed VLC system in a free space transmission length of 3 m, the operated current of 3.5 mA is the optimal control for power-efficiency operation.

**Figure 3:**
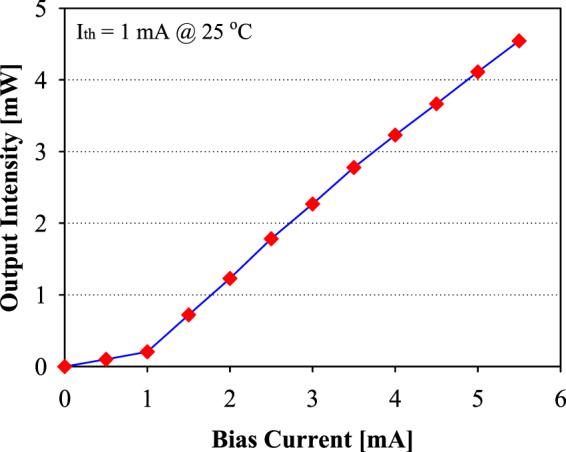
Measured L-I curve of VCSEL laser.

Figure 3 presents the measured plot of optical output (L) as a function of bias current (I_bias_) of 682 nm VCSEL laser at the back to back (B2B) status. The observed threshold current is around 1.0 mA with output intensity of 0.21 mW. As illustrated in Fig. 3, the measured L-I curve is very close to the linear distribution for direct signal modulation. Moreover, the rising and falling times of VCSEL laser are nearly 100 ps. To operate in linear region, the I_bias_ is selected at around 3.5 mA. When the I_bias_ is 3.5 mA, the relative output intensity is 2.78 mW.

### Remodulated OOK Upstream Traffic

The upstream signal is generated by using wavelength remodulation or the downstream signal. In order to produce the upstream signal, the downstream signal is launched into the AOM for OOK upstream remodulation. Here, an electrical pattern generator (PPG) is used to generate the electrical OOK signal, which is applied to the AOM at the client side. The light beam diameter and optical rise time of the AOM are 1.5 mm and 265 ns, respectively. Due to the different data rates and different modulation formats are used, the OOK signal can be modulated directly onto the OFDM downstream signal without the need of downstream signal erasure. Due the limitation of 1.8 MHz bandwidth AOM, 2 Mbit/s OOK format is applied in this proof-of-concept demonstration. Higher upstream OOK data rate could be achieved if higher bandwidth AOM is available in the laboratory. Then, the OOK upstream can be detected by a 50 MHz PIN-PD connected to an electrical error detector (ED) after 1 and 3 m transmission lengths. A 20 MHz Bessel low pass filter (LPF) is used to remove the high frequency OFDM signal and capture the OOK signal. Moreover, to analyze the remodulated OOK upstream performance, the bias currents of VCSEL laser are set at 3, 3.5 and 4 mA with the OFDM downstream signal is present for OOK signal detection.

**Figure 4:**
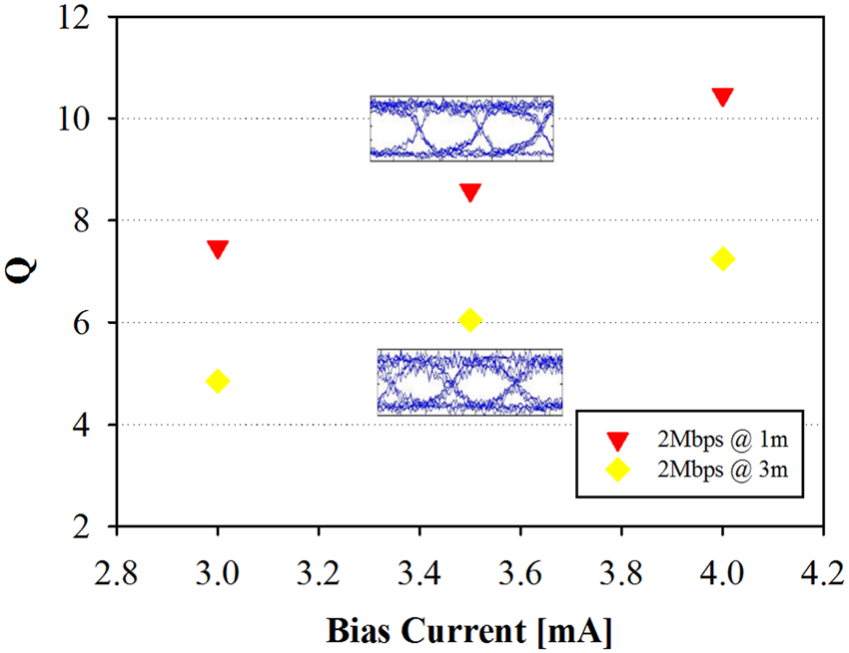
Measured Q factor performance with 2 Mbit/s remodulated OOK format at the bias currents of 3, 3.5 and 4 mA in the free space transmission lengths of 1 and 3 m, respectively. The insets are corresponding eye diagrams.

Figure 4 presents the measured Q factor of the 2 Mbit/s remodulated OOK format under the bias currents of 3, 3.5 and 4 mA in the free space transmission lengths of 1 and 3 m, respectively, when the 20 MHz LPF is used. As illustrated in Fig. 4, when the bias current is increased gradually, the observed Q values are also increased. When the bias current is at 3.5 mA, the Q values of 8.5 and 6.1 are obtained in the free space transmission lengths of 1 and 3 m, respectively. The corresponding BERs are below 10^−9^ at this bias current. Moreover, due to the larger optical SNR in the client side, the measured Q factor after 1 m transmission length is better than that of 3 m long, as seen in Fig. 4. The insets of Fig. 4 present the corresponding eye diagrams at the 1 and 3 m transmission lengths, respectively. The eye diagrams are clear and open. The results show clearly that 10.6 Gbit/s OFDM downstream and 2 Mbit/s remodulated OOK upstream bidirectional VLC after 3 m free space transmission can be achieved. It is also worth to mention that the 3 m free space transmission distance is limited by our optical table; much longer transmission distance can be supported in this scheme.

In this work, 10.6 Gbit/s OFDM downstream signal can be achieved by using only a single VCSEL laser with 3-dB direct-modulation bandwidth of 1 GHz in the central office and a 1.25 GHz bandwidth PIN PD in the client side after a 3 m free-space transmission. The proposed spectral-efficient OFDM-QAM with optimal bit-loading and power-loading algorisms can increase the bandwidth efficiency by 10 times. Besides, a 1.8 MHz AOM is used to re-modulate the OFDM downstream signal to generate a 2 Mbit/s OOK upstream signal in the same transmission length without the need of downstream data signal removal by using a SOA as reported in ref.^29^. The upstream traffic can be increased if a higher bandwidth AOM is available in the laboratory. This is a first demonstration for the remodulated upstream signal in the visible laser-based VLC system.

## Method

### Architecture of Proposed Bi-directional Wavelength Remodulated VLC System

**Figure 5:**
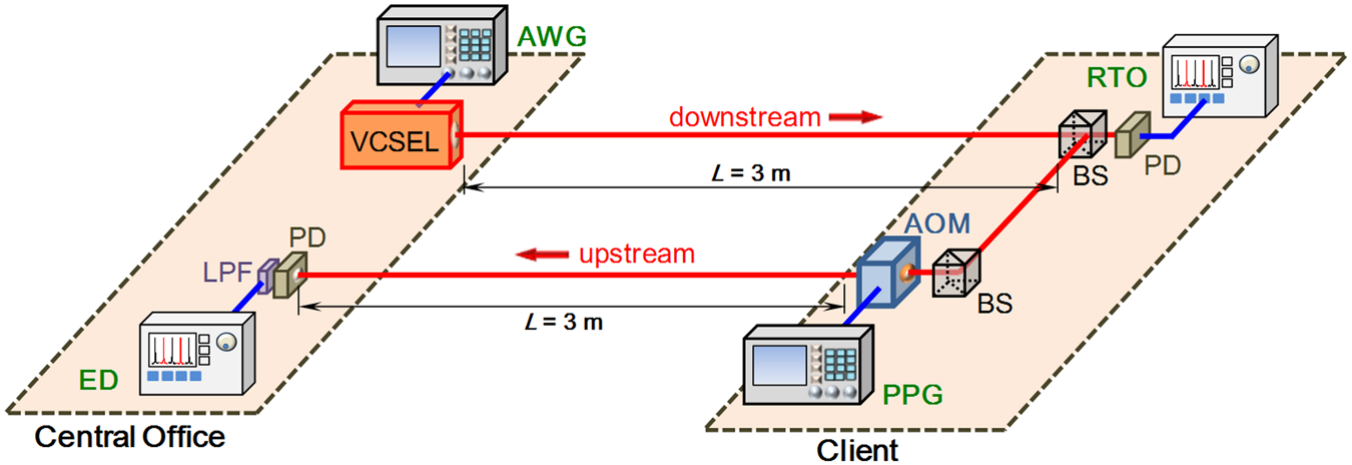
Architecture of the proposed bi-directional VCSEL-based VLC system.

Figure 5 shows the architecture of the proposed VCSEL-based bidirectional VLC system. In the proof-of-concept experiment, at the CO, a 682 nm VCSEL laser with ~1 GHz modulation bandwidth is used to act as a VLC transmitter (Tx) for broadcasting downstream wavelength. Here, the free space transmission lengths are set at 1 m and 3 m. To achieve higher VLC data rate, OFDM-QAM modulation with bit and power loadings are designed and applied to the VCSEL laser via a 2.5 GHz BT circuit. It is worth to mention that our proposed scheme is not a retro-reflecting configuration. As illustrated in Fig. 5, one part of the downstream signal is launched into a 1.25 GHz bandwidth PIN-based photodiode (PD) at the client side. The PD is connected to a RTO for OFDM demodulation. The other part of the downstream signal will act as the seeding light source to be launched into the AOM for the upstream signal generation. The AOM has the 3-dB modulation bandwidth of 1.8 MHz, with the operation wavelength range from 440 to 700 nm. Using AOM for the upstream signal generation can consume a fair amount of power and are bulky. It needs a driving power of 2 W, and its size is 22.4 mm × 74.7 mm × 62.5 mm. It is electrical driven by a PPG to produce the 2 Mbit/s OOK signal. Finally, the upstream signal is detected by a 50 MHz PIN-PD connected to an ED after transmitting 3 m transmission length at the CO.
